# Second Clinical Report on Continued Fever, Based on an Analysis of Forty-Eight Cases

**Published:** 1851-07

**Authors:** Austin Flint

**Affiliations:** Prof. of the Principles and Practice of Medicine, and Clinical Medicine, in the University of Buffalo


### BUFFALO MEDICAL JOURNAL

AND

### MONTHLY REVIEW.

VOL. 7. JULY, 1851. NO. 2

### ORIGINAL COMMUNICATIONS.

*Second Clinical Report on Continued Fever, based on an analysis of forty-eight cases.* By Austin Flint, M. D., Prof. of the Principles and Practice of Medicine, and Clinical Medicine, in the University of Buffalo.

During the summer of 1850 I devoted the leisure hours that I could command to the preparation of a clinical report on Continued Fever, based on an analysis of fifty-two cases, the histories of which I had preserved. This report was published in successive numbers of the Buffalo Medical Journal, from August, 1850, to January, 1851, inclusive. The recurrence of the duties of the winter season rendered it necessary to leave the report unfinished. It remained to treat of the question of the identity of the different types of Continued fever, the subject of diagnosis, and the management of the disease. These topics I proposed to take up during the present summer. In the mean time, during a service of six months at the Buffalo Hospital of the Sisters of Charity, an additional number of cases of Continued Fever have passed under observation, the histories of a large proportion of which were noted, and are preserved. An analysis of the latter will enable me to combine and contrast the results of a similar investigation of the two collections of cases. This is a sufficient inducement to enter upon the labor with reference alone to personal gratification and improvement. Entertaining, however, the hope that the fruits of the investigation may not be devoid of interest for others, and that they may possess some value as contributions to medical science, I propose, under the title of a "Second Clinical Report on Continued Fever," to present such facts and inductions as may be developed by the study of the cases recently collected, considered separately, and in connection with the collection of cases upon which the former report was based. The Hospital records for the six months' service from Oct. 1, 1850, to April 1, 1851, contain the histories of *forty-eight* cases of Continued Fever. This number added to those analyzed during the last summer, will make precisely one hundred cases. The two reports, therefore, will embrace an enumeration and comparison of the events contained in the number of recorded cases of Continued Fever just mentioned.

An hundred cases of Continued Fever, I am aware, will seem but a small number to those who are accustomed to see large hospital wards constantly crowded with persons laboring under this disease. With a view to the interest and perhaps importance of the results of numerical investigations, I could wish that the collections were larger, and I have not been without misgivings lest, on account of the limited amount of data, the time and trouble bestowed upon them might be greatly disproportionate to the fruits they are capable of yielding. But, on the other hand, had my records contained many more cases to be added to those upon which this Report is based, the labor of the analysis, enumerations, and comparisons would wholly preclude the undertaking. It is not improbable that in the overplus of materials may consist the chief obstacle in the way of such investigations by some of those whose opportunities for observation are much more extensive than mine. When Louis resolved to spend a series of years in the collection of data for numerical analysis, he not only relinquished the duties of private practice, but his mind was relieved even of the responsibility of directing the management of the diseases, the phenomena of which it was his sole business to record. And when he entered upon the task of classifying, enumerating and comparing the facts which he had gathered in the wards of *La Charite*, he intermitted the labors of observation and registration, and, retiring into the country, devoted his whole attention, for many months, to analytical researches. Few persons would be satisfied thus to consecrate their time and energies to purely scientific toil, and of those inspired by a similar zeal in behalf of medical science, how few are able to divest themselves of the many daily cares and duties which almost of necessity occupy a large share of the exertions of mind and body!

Small, comparatively, as is the number of cases I have collected, and less complete, in some respects, than could be desired, as are the histories, I venture to think that they may furnish results not altogether valueless. Without the encouragement of such an expectation, it would, assuredly, be worse than folly to undertake a task which will consume the leisure hours of several weeks. Should the Report repay but poorly the trouble of perusal, it may perhaps moderate a disposition to complain, to consider that its preparation has cost vastly more labor than will be requisite to discover its demerits, and that the reader is therefore much less unfortunate than the author.

The histories of the cases upon which the former Report was based, were recorded wholly by myself. It is proper to state that this is not true of the cases in the present collection. In a considerable proportion of the latter, the recording of symptoms and events was entrusted to Mr., now Dr. Sandford Eastman, at that time a resident student at the Hospital. The patients were daily examined, and the notes revised by me. In several particulars the daily records are more full than would have been practicable had the whole duty been performed by myself, but in some points they are probably less so. In the accuracy of the events and symptoms registered by Dr. E., I have entire confidence, and I avail myself of this occasion to express my indebtedness to his industry and fidelity.

Of the forty-eight cases forming the subjects of the present analysis, *twenty-nine* presented unequivocal evidences of the *Typhoid* type of Continued Fever; *ten* were considered, as unequivocally, cases of *Typhus*, and *nine* are classed under the head of cases of *doubtful type;* under the latter head are included all the cases in which there might be some ground of uncertainty as to the correctness of the diagnosis, had they been placed in either of the preceding classes. The reasons for this uncertainty in a certain proportion of the cases of Continued Fever which fall under observation, will be considered hereafter, in connection with the subject of diagnosis.

Of the *twenty-nine* cases of *Typhoid* fever, *seven* proved fatal. *Four* of the *ten* cases of *Typhus* ended fatally. All of the *nine* cases of *doubtful type* recovered. *Eleven*, thus, of the whole number of cases, i. e. 48, were fatal. The number of deaths is precisely the same as in the first collection of cases, making *twenty-two*, out of the *one hundred cases.*

I shall arrange the facts and considerations to be submitted in the following Report, in the same divisions before adopted, assigning to each division a separate section. In many of the points of investigation, the interest and importance of the results are restricted to those cases which are considered to belong either to the *typhoid* or *typhus* type of continued fever. In all such instances there will be no advantage in referring to the cases of doubtful type. It should be added, that in this, as in the former collection, the uncertainty as to the diagnosis of the cases of *doubtful type* concerns solely the question as to which of the two types, typhus or typhoid, they belong. No doubt exists as to their having been cases of continued fever.

For convenience of reference to corresponding portions of the first Report, the number and page of the sections will be prefixed to the sections of the present Report having the same headings.

SECTION FIRST.

*Age, Sex, Occupation, Civil Condition, Nativity, Habits, Season, Constitution and previous Health of the Patient. Period of residence in this Country and in this City. Duration of the Disease before coming under Observation.* [No. for August, 1850. Page 136.]

The ages of the patients are given in *twenty-six* cases of the *typhoid* type, and are respectively as follows:

| | | | | |
|---|---|---|---|---|
| 50, | 38, | 35, | 35, | 28, |
| 27, | 26, | 24, | 24, | 23, |
| 22, | 21, | 20, | 20, | 20, |
| 20, | 19, | 18, | 18, | 18, |
| 17, | 17, | 15, | 14, | 12, |
| 9, | | | | |

Average age 22 9-13.

This differs in a fraction only from the mean obtained in the former analysis, which was 22 2-17. The coincidence developed by the two analyses is in accordance with the law of the disease, as respects age, stated in the first Report.

Of the *typhus* cases, the ages are given in *eight*, and are as follows:

| | | | | |
|---|---|---|---|---|
| 54, | 26, | 25, | 24, | 21, |
| 19, | 17, | 24, | | |

The maximum, and the mean are higher than in the typhoid cases. The average is 26 1-4. In the first Report it was found, in *ten* cases, to be 26 1-2. Here, too, is the difference of a fraction only—a coincidence striking, and also in accordance with a law of the disease. Comparing the average age of the fatal cases, with the average of the whole number, the following is the result:—In *six* fatal cases of *typhoid* the average age was 31. The patient with the maximum age (60) died. The law that the average age of those who die with this form of the disease, is greater than the average in favorable cases, is here exemplified, as it was in the former analysis, but in a more striking degree in the present collection. The age of a patient affected with typhoid fever is thus of some account in the prognosis.

Of the *typhus* cases, the average age in three of the fatal cases was 24 1-3, being less than the average in the cases that recovered.

*Sex.*—Of the forty-eight cases, *thirty-three* were males, and *fifteen* females. Of the twenty-nine *typhoid* cases, *twenty-one* were males, and and *eight* females. Of the ten *typhus* cases, *seven* were males, and *three* females. Of the cases of *doubtful type*, *five* were males, and *four* females. The relative number of females is considerably greater than in the first collection of cases.

*Occupation.*—In the histories of the twenty-nine *typhoid* cases, the occupations noted are as follows:—laborers, fourteen; domestics, (females,) *six;* cook, (male) 1; porter in hospital, fireman, boatman, wheelwright, and farmer, each *one* case. In *three* cases the patients were too young to have any occupation. In *nine* of the *typhus* cases the occupations were as follows:—laborers *five*; boatman, domestic, Sister of Charity, candidate for the Sisterhood, one case of each.

The patients, as will be perceived, were mostly of the class, in social position, who would be expected to resort to a charitable institution for the relief of the sick.

*Civil Condition.*—Of *twenty-eight* cases of the *typhoid* type in the histories of which the civil condition of the patients is noted, *twenty-six* were single, and *two* married.

Of *nine* cases of *typhus*, in *eight* the condition was single, *one* patient only being married.

*All* the *nine* patients with continued fever of doubtful type were single.

The great preponderance of the cases in which the patients were single, is probably to be explained, first, by the fact that the disease, more especially the typhoid form, affects in preference the young; and, second, a very large proportion of all the patients who are admitted into the hospital with different diseases are unmarried, and are led to seek admission in sickness in consequence of not having homes of their own.

*Nativity.*—*Typhoid cases: nineteen* were Irish; *five* Germans; *three* Englishmen; *two* Americans.

*Typhus cases: eight* were Irish, and *two* Americans. In the two latter cases the disease was contracted within the Hospital. It is worthy of notice, that in all but the two cases in which the disease was contracted at the hospital, the patients affected with *typhus* were from Ireland. Nearly the same results obtained in the first collection.

*Habits.*—The habits of the patients as respects intemperance, etc., were generally not ascertained.

*Season.*—Of the twenty-nine *typhoid* cases, in *one* the commencement of the disease is dated in September, three days before my service commenced. The remaining twenty-eight cases occurred as follows:

In October, *eleven* cases;
In November, *eight* cases;
In December, *eight* cases;
In January and February *no* cases;
And in March *one* case.

The predilection of this type of continued fever for the months of October, November and December is thus exhibited. It may be suspected that the above facts are owing in part to the diminished influx of immigrants during the winter, inasmuch as it will presently appear that a large proportion of the patients had recently arrived in this city. That this explanation is not applicable is shown by the Hospital Register during the summer season when the floating population of the city is largest. From April to September, inclusive, 1850, I find during the six months, respectively, the number of cases recorded as typhoid fever to have been as follows:—April, May, and July, in each, *two* cases; August, *five* cases; September, *two* cases. In all, *thirteen* cases.

The ten cases of *typhus* occurred as follows:

September 30th, *one* case;
October, *one* case;
December, *two* cases;
March, *five* cases;
April, *one* case.

During the six months from April to September, inclusive, I find the following cases of *typhus* registered:

April, *two* cases;
May, *four* cases;
June, *one* case;
July, *three* cases;
August, *three* cases;
And September, *two* cases.
In all, *fifteen* cases.

These results accord with the more extended observations from which it has been deduced that while *typhoid* fever is much more liable to occur in the autumnal, than in the other months of the year, *typhus* occurs irrespective of season.

*Constitution and Previous Health of the Patients.*—The constitution and previous health are not noted in the histories of *seven* of the *typhoid* cases. Of the remaining *twenty-two* cases, in *twenty* the patients possessed good constitutions, and were attacked while in excellent health. In *one* case, the patient, a porter in the hospital, was laboring under a chronic ulcer of the leg of long standing; and in *one* case the patient had chronic conjunctivitis.

Of the *typhus* cases, the histories are defective, in these points, in *four*. In all the remaining *six* cases, the constitution and general health were good. Of the cases of *doubtful type*, in a single case only is it stated that the patient was not in perfect health. In that case the patient was attacked with fever in the hospital, having entered for, and still laboring under chronic conjunctivitis. In the remaining cases, save one, the history of which is silent on the subject, the health and constitution were good.

So far as these results go, they concur with those presented in the first Report in leading to the conclusion that persons having good constitutions, and in good health, are those most likely to be attacked with continued fever.

*Period of Residence in this Country and in this City.—Typhoid Cases.* The facts pertaining to these points are noted in the histories of *twenty* cases, and are presented in the following table:

| | *Period of Residence in Country.* | *Do. in City.* |
|---|---|---|
| 1. | Nine months. | Two months. |
| 2. | Four months. | One week. |
| 3. | Two and a half weeks | Entered day of arrival. |
| 4. | Twelve months. | Two months. |
| 5. | Three months. | Ten days. |
| 6. | Sixteen months. | Sixteen months. |
| 7. | Five weeks. | Four weeks. |
| 8. | Two weeks. | Entered day of arrival. |
| 9. | Six months. | Four months. |
| 10. | Four weeks. | Not stated. |
| 11. | Four years. | Two months. |
| 12. | A few months. | Entered day of arrival. |
| 13. | Eight months. | Eight months. |
| 14. | Twenty-one months. | Twenty months. |
| 15. | Two weeks. | Four days. |
| 16. | Two weeks. | Four days. |
| 17. | Five weeks. | Two weeks. |

| | |
|---|---|
| 18. Recently arrived. | Recently arrived. |
| 19. Four weeks. | Three and a half weeks. |
| 20. Four weeks. | Three and a half weeks. |

In the *nine* remaining cases the histories are defective as respects the points under consideration. It is not noted in a single case that the patient was a permanent resident of the city.

It will be apparent on reference to the foregoing table, that this, as well as the first collection of cases, affords a striking illustration of the law developed by the researches of Louis, viz., that typhoid fever is prone to affect those "placed in circumstances entirely new to them," which are incident to recency of residence. It may be said that the inmates of an hospital in a thoroughfare like Buffalo, would be expected to consist chiefly of persons lately arrived, they being far more likely to resort to such an institution than the settled inhabitants of the place. It is true that a large proportion of the patients admitted with all diseases are of the former class; but the preponderance is not nearly so great in the aggregate of other affections than typhoid fever. In this connection it should be stated, that typhoid fever, although occurring occasionally among the fixed population of this city, is by no means a common disease, and, until within a few years past, was almost unknown. During the six months that the cases in this collection were recorded, it was by no means prevalent, unless among the same class of persons, recently arrived, which furnished the cases at the hospital. My private practice not embracing many patients of this class, I am unable to say to what extent it prevailed among them. The cases coming under my observation, aside from those in hospital, during the period first mentioned, included only two or three cases, excepting a few cases of infantile continued fever, which perhaps would appropriately be called typhoid.

*Typhus cases.*—Of all but *two* of the ten cases of *typhus*, the histories contain information on these points, which is presented in the following table:

| *Period of Residence in Country.* | *Do. in City.* |
|---|---|
| 1. Five weeks. | Not stated. |
| 2. Five weeks. | Five days. |
| 3. Eight days. | Three days. |
| 4. Eleven days. | Three days. |
| 5. A few days. | Ten days. |
| 6. Eight days. | Three days. |
| 7. Resident in Hospital. | |
| 8. Do. Do. | |

Taking into consideration the infrequency of continued fever of the typhoid type, and the still rarer occurrence of typhus, in this place, except among foreign immigrants, it seems probable that the remote special cause of the disease was generally imbibed prior to the period of residence in this city, if not prior to arriving in this country.

*Duration of Disease before coming under Observation.*—In none of the cases, except those originating within the hospital, were the patients under observation from the very commencement of illness, and the duration before admission presents considerable diversity in the different cases. In several instances the patients had been ailing for several days, but had kept up until they entered the hospital, but in most cases they had been confined to the bed for one, two, three, four or more days. The variations, as respects this point, are obviously due, in a great measure, to circumstances extrinsic to the disease. More or less delay in getting admission might arise from various causes, and patients would wait, some longer, and some for a less period, before seeking to be admitted. For these reasons, there would be no object in presenting the facts pertaining to the duration of the disease before coming under observation, contained in the histories of the individual cases.

There is one fact, however, relating to this point which is worthy of notice. The duration of the disease before the patients entered the hospital, as a general remark, is strikingly shorter in the cases of *typhus*, than in the cases of *typhoid.* In *one-half* the cases of typhus it is stated that the patients did not take to the bed prior to entering the hospital; and of the remaining *five* cases the histories are defective in information on this point in *three;* in *one* the patient took to the bed the day before admission; and in the other case the patient was attacked in hospital.

Of the twenty-nine cases of typhoid, it is stated that the patients had not taken to the bed before admission, in only *five* cases!

The fact that patients with *typhus* enter earlier in the disease than those affected with the *typhoid* type of continued fever, is probably significant of another fact, viz., that the onset of the former impresses the patient more strongly with a sense of greater gravity of the malady.

### SECTION SECOND.

*The Access, its Duration and Symptoms. Circumstances supposed to have been concerned in the Production of the Disease.* [Vol. VI., No. for August, page 143.]

*Duration.*—The access, in other words the prodromic, or forming stage of continued fever, embraces the period which elapses from the commencement of ailment to the time when the disease is established, or the febrile career fairly entered upon. But the symptoms of this period generally merge so imperceptibly into those which follow, that it is often extremely difficult to draw the line separating the former from the latter. And especially to determine the time when the access ended, and the fever began, in cases which do not come under observation until after the latter epoch, would generally be impracticable, unless some circumstances be fixed upon which shall serve as a sign or criterion of the point which we wish to establish. In my former Report it was suggested to select as the juncture at which the commencement of the febrile career should be dated, the day on which the patient, from the amount of debility, etc., feels no longer able to sit up, but is compelled to take to the bed. This is an arbitrary rule, not claimed to be always accurate, but it may be doubted if any other uniform test can be suggested which, in the average of cases, is more reliable; and it has the advantage of being readily and exactly determinable in the great majority of instances. It will seldom happen that we cannot ascertain, either from the patient, or from others, how many days have been spent in bed. With an equal amount of debility, etc., some patients will take to the bed sooner than others. Various circumstances, moreover, may prevent patients from yielding to an inclination to take to the bed, and stimulate them to make strong exertions to keep up, and even continue at labor as long as possible. Some of the patients in this, and the first collection of cases, had not only kept about, but had rode many miles, or walked a considerable distance to the hospital, when the presence of the eruption, and other symptoms, showed that the fever had been established already several days. Other patients differently situated, or of a different mental constitution, take to the bed on the first indications of ailment. These cases, it may be presumed, will compensate for each other, so that, in the long run, the event fixed upon will be found to be sufficiently precise for all practical purposes, and, at all events, as much so as any other test equally available.

In the histories of a large proportion of the cases forming the present collection, it is not definitely stated how long the patients had been confined to the bed. This omission was owing to inadvertence. In *ten* cases of the *typhoid* type in which the facts with respect to this point were ascertained, the duration of the access was as follows:—

| | |
|---|---|
| Four days, - - - - | Three cases. |
| Three days, - - - - | Three cases. |
| Six days, - - - - | One case. |
| Attack sudden, - - - - | Three cases. |

The period from the first symptoms of ailment is given in the histories of most of the cases, but the defect is in not stating how long, if at all, the patients had kept their beds. The foregoing cases suffice to show that the access is sometimes sudden, but in other cases, having a duration varying from three to six days.

In the histories of *typhus* cases the duration of the access is specified as follows:—

| | |
|---|---|
| Three days, - - - - | Two cases. |
| Two days, - - - - | One case. |
| Four days, - - - - | Two cases. |
| Attack sudden, - - - - | One case. |

In two of the remaining cases it is noted that the duration was 'several days.'

The foregoing results do not afford any confirmation of the law, deduced from extensive observations, that the *typhus* type of continued fever oftener commences with a sudden attack, or is ushered in by an access of shorter duration than *typhoid.* The cases, however, are too few to render the comparison in this particular of much value.

*Symptoms of Access.*—In all save a very few cases, the symptoms of the access were not observed, the patients not entering the hospital until this period was passed, and more or less progress made in the febrile career. Information respecting the previous history was generally obtained from the patients themselves—a source not always perfectly reliable, owing to the condition of mind incident to continued fever. The replies to questions may be coherent and rational, but nevertheless incorrect, while perhaps there are no manifestations of delirium, nor any deliberate intention on the part of the patient to deceive. Of the more prominent of the early events that mark the access, however, most patients retain a clear recollection, certainly during the early part of the febrile career, and comparatively few entered the hospital at an advanced period of the disease, when the reliability of their statements would be invalidated by the mental condition just referred to. In some of the cases an account of the symptoms of the access is defective, or wholly wanting, because the patients were plainly incompetent to give information on the subject. The questions generally were confined to the more prominent events, such as cephalalgia, chills, diarrhœa, etc. The records are not uniformly so full with respect to these as could be desired.

*Chills.*—In *twenty-one* cases of the *typhoid* type, the histories state distinctly that chills were, or were not present. And of this number of cases one or more chills marked the access in all but *one* case, i. e. in *twenty cases.* In one case, it is stated, no chill occurred. The degree of chill, and its duration, and whether rigor accompanied, are not stated. In *four* cases, it is stated, a recurrence of chill was experienced, and in *two* of these cases, a *third* chill took place.

With respect to an increase of heat after the chill, sensible to the patient, or rather sufficient to attract his attention, positive information is contained in the histories of *fifteen* cases. It occurred in *twelve* of these cases, and in the remainder, *three*, was not noticed.

Perspiration occurred after the chill in *ten* of the *fifteen* cases the histories of which contain information on this point. In *five* no perspiration followed.

Of the ten cases of *typhus* the symptoms of the access were not recorded in the histories of *five*. This proportion of cases in which the records are thus defective, is probably significant of the fact that the mind, earlier, and more uniformly, becomes affected in this type of continued fever, so as to render information concerning the previous history more uncertain, or not reliable.

Of the *five* cases in which an account is given of the access, a chill occurred in *four*, and in the remaining cases it is not stated whether this symptom occurred or not. A recurrence of the chill took place in *one* of the *four* cases. It is stated that the chill was followed by increased heat in *two* cases; and of these, perspiration succeeded in *one*, and it did not follow in the other case.

*Cephalalgia.*—Of the *typhoid* cases it is stated positively with respect to this symptom in *twenty-three.* Cephalalgia occurred in *twenty-one* of this number, and was absent in *two* cases.

Of the *typhus* cases, it is stated positively in *six.* Cephalalgia occurred in *four* of this number, and was absent in *two.* In the histories of some cases this symptom is said to have been severe or intense, but generally the degree of pain is not expressed.

*Pain in the loins*, is stated to have been present in *fourteen* cases of the *typhoid* type. It is stated to have been absent in *two* cases. Of the remaining *thirteen* cases it is presumed to have been absent in several, at least, from the fact that it would probably have been included in the previous history had it been present.

Of the *typhus* cases the presence of this symptom is affirmed in *one* case only, and it is stated to have been absent in *two* cases. In *seven* the histories do not contain information on this point.

*Pain in limbs*, is stated to have been present in *sixteen* of the *typhoid* cases. The histories of the remaining cases are silent with respect to this symptom.

It is stated to have been present in *two* cases of *typhus*, and nothing stated on the subject in the remaining cases.

The patients were not questioned with respect to a symptom which, as all observers are aware, is a common attendant on an attack of fever, viz, lassitude, or a painful sense of fatigue.

*Nausea and Vomiting.*—In the histories of *fifteen typhoid* cases, it is stated positively with respect to these symptoms. They were present in *twelve* of that number, and in *one* case nausea existed without vomiting. Generally these symptoms were slight. In *two* of the *fifteen* cases they were not present.

In the histories of the *typhus* cases it is only recorded that in *one* case there was no nausea nor vomiting. Nothing is stated on the subject in the remainder.

*Diarrhœa.*—The histories of the *typhoid* cases contain information respecting this symptom in twelve. Of this number, it was present in *seven*, and absent in *six*. It is to be presumed that it was not a prominent symptom, to say the least, in the cases, the histories of which are silent with respect to it.

It was absent in *six* of the cases of *typhus*, and is not stated to have been present in a single case of this type.

This symptom might have been present in some cases in which it is stated to have been absent, if simply looseness of evacuations, without increase in number, were considered to constitute diarrhœa, after the custom of the French. It would not be stated that diarrhœa had existed except in cases in which the dejections were more or less frequent.

*Thirst* is stated to have been present in *twelve* cases of *typhoid* and *two* cases of *typhus*. Its absence is not noted in the history of a single case of either type. In a large proportion of the cases in the histories of which the presence of thirst is noted, it is stated to have been intense.

*Anorexia* is stated to have been present in *twelve* cases of *typhoid*, and in *four* cases of *typhus*. Its absence is not noted in a single case of either type.

*Cough* is not mentioned as a symptom of the access save in *two* cases of *typhoid*, and *one* case of *typhus*.

*Epistaxis* is noted to have occurred in the histories of *three typhoid* cases; and its absence is noted in *five* cases of the same type. It is not noted to have been present in any case of *typhus*, and its absence is noted in only a single case.

*Causation.*—The patients were generally asked if they could attribute their illness to any particular cause or causes; if they had ever before had an attack of fever; and, if immigrants, whether any disease prevailed in the vessels which brought them to this country, or at the places of their residence about the time of their leaving.

The histories of *twenty* of the *forty-eight* cases contain nothing relative to either of the points just mentioned. The facts pertaining to the remaining *twenty-eight* cases, which are noted, are as follows:

In *two* of the *typhoid* cases the patients said the disease commenced with their taking cold. The same was stated by *one* of the patients affected with fever of a *doubtful type.*

In *six typhoid* cases the patients, immigrants, declared that there was no sickness, but sea sickness, on board the vessel in which they came over, nor at the places from which they came. The only exception to this statement is, that one patient said there was one person ill on board the vessel, but he did not know with what disease. In the histories of those cases of *typhus* in which the patients were immigrants, nothing is stated relative to sickness on the voyage, or before sailing. In no case of either type is it stated that the patient had been in proximity to persons laboring under fever. These facts, in so far as they go, while they show the liability of immigrants to be attacked with fever, shortly after their arrival, and, considering the infrequency of continued fever in this city, render the supposition probable that the special cause of the disease, in the cases referred to, was introduced within the system prior to their reaching this country, do not supply any illustration of the agency of contagion in the production of the disease.

In the history of *one* case of *typhoid* it is stated that the father and mother of the patient were, at the same time, in the hospital with continued fever, the father being affected with *typhus*, and in the case of the mother the disease being of *doubtful type.* In *two* of the *typhoid* cases the patients were brothers, both cases being in progress at the same time.

In *two* cases of *typhus* the patients came over in the same vessel, both cases progressing at the same time.

In *two* other cases the patients came over in the same vessel, one case being of *typhus*, and the other of *doubtful type.*

In *one* case of *typhus*, and *one* of *doubtful type*, the patients were brother and sister, and came over in the same vessel.

These facts probably show community as respects exposure to the special remote cause of the disease.

In *one* case of *typhus* the patient stated that he had not changed his linen, or any of his clothing, from the time he embarked in Ireland, up to the date of his admission into the hospital!

*One* patient affected with fever of *doubtful type* stated that he had been accustomed to sleep in a small room in which were lodged from fifteen to twenty persons.

In not one case of *typhoid* does the history state that the patient had ever before had fever. In this case the patient stated that he had had fever a year before in Ireland, both his parents being affected with the disease at the same time. In *one* case of *typhus* the patient said he had had fever twenty-five years before. In *two* cases of *doubtful type* the patients said they had previously had fever, one three years, and the other two years before. The small proportion of cases in which the previous occurrence of fever is stated, is obviously the point most worthy of note, and it is to be remarked that in these cases it is by no means certain that the disease referred to was continued, or indeed any form of essential fever.

In several of the cases the probable source of the disease was in contagion. I will give the circumstances favoring this view of the causation in the cases referred to, respectively. The cases were of the following types: *typhoid, three* cases; *typhus, two* cases. In all *seven* cases.

Of the *typhoid* cases, in one case the patient, four or five weeks before being attacked, was with a brother affected with fever. He had slept with him at night, and labored during the day time. In another case the patient, who had lately come to this country, a short time before being attacked, (the time is not definitely stated,) had taken care of a friend affected with fever who came over in the same vessel. In the third case the patient was employed as a porter in the hospital. He was in the general ward much of the day time, and slept in the ward at night. There had been a short time before he was attacked several fever patients in the ward, and he had had particular charge of them at night. He had had a chronic ulcer of the leg, and had also had erysipelas. The ulcer had healed about two months before he was attacked with fever.

Of the *typhus* cases, in one, the patient was a *Sister of Charity* attached to the hospital. She was attacked while nursing in the general ward in which there had been cases of fever of both the typhus and typhoid types. In the other case, the patient was a candidate for the Sisterhood, and was attacked while rendering services in the same ward.

Of the cases of *doubtful type*, in one, the patient was a surgical patient laboring under chronic conjunctivitis, who was frequently in the general ward in which fever patients were received. In the other case the patient was one of the *Sisters of Charity* who was assigned to the general ward, and had lately nursed fever patients affected with the typhus and typhoid form of the disease.

Adding together the cases in the present and former collection in which the causation of the disease appeared to involve contagion, the sum is *ten*, i. e. *ten* of the *one hundred* cases.

The cases most clearly due to contagion are those in which the patients were *Sisters of Charity.* In the former Report it was stated that the two Sisters to whom had been assigned the care of nearly all the fever cases during the winter, alone, of all the inmates of the hospital, became affected with fever. The two Sisters, and the candidate for the Sisterhood, included in the present collection of cases, had also special care of fever cases, and of the Sisters to whom other duties were assigned than nursing fever patients, none have been affected with fever. *There have been ten Sisters of Charity attached to the hospital during the past two years, and of these the four that have had continued fever have been those who have been especially brought into contact with fever patients.*

An interesting circumstance connected with these cases is, that they all occurred during the Lenten season, as also the case in the person of the candidate for the Sisterhood. The exposure was not greatest at this portion of the year, nor were the duties more arduous, and therefore it seems fair to suppose that the abstinence, and increased devotional exercises of this period must have had some agency in the causation of the disease, rendering, probably, the system less able to resist, or eliminate the miasm of contagion.

*To be Continued.*

